# Possible Paternal Hepatitis B Virus Infection with Different Clinical Courses between Siblings: A Report of Two Cases

**DOI:** 10.1155/2022/5812135

**Published:** 2022-03-07

**Authors:** Yoshiaki Sasaki, Hiroki Kajino

**Affiliations:** Department of Pediatrics, Abashiri-Kosei General Hospital, Abashiri, Hokkaido, Japan

## Abstract

The incidence of hepatitis B virus (HBV) infection is expected to decrease in the future owing to the preventive measures adopted against mother-to-child transmission of HBV and implementation of universal HBV vaccination for children. However, no countermeasure has been established against horizontal infection in nonvaccinated children. We report the case of two siblings who had different clinical courses of possible paternal HBV infection. The younger sibling developed acute hepatitis, whereas the older sibling was an asymptomatic HBV carrier. To eradicate HBV, HBV vaccination of all children and HBV infection screening of fathers and other family members should be encouraged.

## 1. Introduction

The incidence of hepatitis B virus (HBV) infection is expected to reduce in the future with the implementation of prevention projects against mother-to-child transmission of HBV and universal HBV vaccination [1]. In addition, the development of antiviral drugs for HBV has contributed to the reduction in the infection rate [1]. However, to date, no countermeasure has been established against horizontal infection, such as paternal infection of HBV in nonvaccinated children. Here, we report a case of possible paternal HBV infection that resulted in different clinical courses of HBV infection between siblings.

## 2. Case Presentation

### 2.1. Case 1

A 1-year and 4-month-old girl, with an unremarkable birth and medical history presented with vomiting of 3 days and diarrhea of 2 days before being admitted to our hospital with a diagnosis of acute rotavirus gastroenteritis. Her laboratory findings on admission showed severe liver dysfunction, with an alanine aminotransferase (ALT) level of 583 U/L and aspartate aminotransferase (AST) level of 406 U/L. Simultaneously, the tests for hepatitis B surface antigen (HBsAg) and immunoglobulin *M* antibody to hepatitis B core antigen were positive. Therefore, she was diagnosed with acute hepatitis B. She had not been vaccinated against HBV. With conservative treatment, such as rest and hydration, her symptoms improved without a fulminant course. She was discharged on the 10th day of admission. Three months afterwards, her test results revealed seroconversion of HBeAg/HBeAb. Eventually, at 8 years of age, she was declared HBsAg-negative, and virological remission was achieved (Table 1).

### 2.2. Case 2

The second patient, a 2-year-7-month-old girl, was the older sibling of the first patient. She was born with a low birth weight of 2,272 g and had no remarkable medical history. She was not vaccinated against HBV. Although she had no symptoms when the first patient was diagnosed with acute hepatitis B, her laboratory findings revealed mild liver dysfunction, with an ALT level of 87 U/L and an AST level of 79 U/L. She was HBsAg-positive, hepatitis B envelope antigen (HBeAg)-positive, and hepatitis B envelope antibody-negative. Her HBV DNA (PCR) was high (>8.2 log IU/L), and the HBV genotype was type B. Simultaneously, she was diagnosed as being an HBV carrier. Subsequently, she was followed up for inactive chronic hepatitis, and seroconversion of HBeAg/HBeAb was detected at the age of 3 years and 8 months (Table 2). To date, she is being followed up at an outpatient clinic as an inactive carrier of HBV. When the first patient developed acute hepatitis, her father was HBeAg-positive, whereas her mother was HBsAg-negative and antihepatitis B surface antibody-positive. Therefore, the HBV infection was considered to have been transmitted to the siblings from their father who was an asymptomatic HBV carrier; however, the actual source of the infection was unknown.

## 3. Discussion

In this report, we observed different clinical courses of horizontal HBV infection in siblings, transmitted from their father possibly. HBV is detected not only in blood but also in urine, saliva, nasopharynx, and tears, which may be sources of infection [2]. Therefore, the risk of HBV horizontal infection in daily life exists. In the literature, the prevalence of HBsAg carriage among children of 1 year or older has been reported to be higher than that among those aged <1 year [3]. This pattern of age distribution suggests that horizontal infection is an important route of HBV infection during early childhood [3]. Vaccine failure leading to mother-to-child infection is the major cause of chronic HBV infection in Japanese children, with paternal infection being the second most common mode of transmission [4]. Although the incidence of paternal infection has been reported to be lower than that of mother-to-child infection, 19.2% of HBeAg-positive fathers have been reported to transmit HBV infection to their children [5]. In our report, the HBV genotype of the siblings' father was not examined, but we diagnosed that the route of HBV transmission of the siblings was paternal, as no other HBV-infected person, except their father, was detected as a possible contact. Infants are susceptible to chronic persistent HBV infection owing to poor cytotoxic T cell (CTL) response [6]. Therefore, the CTL response of the second patient might have been weaker than that of her sibling. In addition, genetic variants in the HLA-DP locus, including HLA-DPA1 and HLA-DPB1, are associated with the chronicity of hepatitis B in literature [7]. Therefore, the second patient may have genetic variants in the HLA-DP gene.

To prevent mother-to-child HBV infection, HBV screening and preventive measures for pregnant women have been developed. However, HBV screening of fathers and other family members is necessary to prevent horizontal infection [8]. The World Health Organization recommends universal vaccination to eradicate HBV [1]. Despite routine immunization of infants with the HBV vaccine being initiated in 2016 in Japan, many children remain unvaccinated. Therefore, we must remember that many children do not benefit from the HBV vaccine program at this time, especially those who are not within their routine vaccination age.

In conclusion, we reported cases of possible paternal HBV infection that resulted in varying clinical presentations of HBV infection in the offspring. Our findings suggest that we should encourage HBV vaccination of all children and screening of HBV infection for all family members, including fathers. These measures are expected to minimize horizontal infection of HBV.

## Figures and Tables

**Table 1:** Laboratory results of the first patient.

	HBsAg	Anti-HBs	IgM anti-HBc	HBeAg	Anti-HBe	HBV DNA (log IU/L)	ALT (IU/L)	AST (IU/L)
Age	‒	‒	‒	‒	‒	‒	‒	‒
1 year and 4 months	Positive	Negative	Positive	Positive	Negative	>8.2	583	406
1 year and 7 months	‒	‒	Negative	Negative	Positive	N.D.	11	29
8 years and 0 month	Negative	Positive	‒	‒	‒	N.D.	13	25

HBsAg, hepatitis B surface antigen; Anti-HBs, antihepatitis B surface antibody; IgM anti-HBc, immunoglobulin M antibody to hepatitis B core antigen; HBeAg, hepatitis B envelope antigen; Anti-HBe, antihepatitis B envelope antibody; HBV DNA, hepatitis B virus-deoxyribonucleic acid; ALT, alanine aminotransferase; AST, aspartate aminotransferase; ‒, no result available; N.D, not detected.

**Table 2:** Laboratory results of the second patient.

	HBsAg	Anti-HBs	IgM anti-HBc	HBeAg	Anti-HBe	HBV DNA (log IU/L)	ALT (IU/L)	AST (IU/L)
Age	‒	‒	‒	‒	‒	‒	‒	‒
2 years and 7 months	Positive	Negative	Negative	Positive	Negative	>8.2	87	79
3 years and 10 months	‒	‒	Negative	Negative	Positive	N.D.	44	55
15 years and 10 months	Positive	Negative	Negative	Negative	Positive	3.27	10	17

HBsAg, hepatitis B surface antigen; Anti-HBs, antihepatitis B surface antibody; IgM anti-HBc, immunoglobulin M antibody to hepatitis B core antigen; HBeAg, hepatitis B envelope antigen; Anti-HBe, antihepatitis B envelope antibody; HBV DNA, hepatitis B virus-deoxyribonucleic acid; ALT, alanine aminotransferase; AST, aspartate aminotransferase; ‒, no result available; N.D, not detected.

## Data Availability

The data used to support this study are included within this article.
